# A qualitative investigation of leisure benefits for social and psychological health among international volleyball players living in South Korea

**DOI:** 10.1080/17482631.2022.2131216

**Published:** 2022-10-06

**Authors:** Young Ik Suh, Junhyoung Kim, Sanghak Lee, Sua Han, Se-Hyuk Park

**Affiliations:** a Department of Sport Management, Wellness, and Physical Education, University of West Georgia, Carrollton, GA, USA; b Department of Health & Wellness Design, Indiana University, Bloomington, IN, USA; c School of Business, Korea Aerospace University, Seoul, South Korea; d Department of Sport Sciences, Seoul National University of Science and Technology, Seoul, South Korea

**Keywords:** Acculturation, leisure, social health, mental health, stress-coping

## Abstract

**Purpose:**

The proportion of foreign players in global sports industries has steadily increased in the last decade. This qualitative study aims to capture the benefits of leisure activities for the social and psychological health of international volleyball players affiliated with South Korean volleyball leagues.

**Method:**

A purposeful criterion sampling strategy was employed in this qualitative study. Findings: Based on semi-structured interviews with 12 participants, in this study three major themes pertaining to leisure benefits are identified: (a) new leisure opportunities and coping, (b) inter-intra group friendships, and (c) acculturation.

**Conclusions:**

These benefits can contribute to social and psychological health for the participants. Practical implications of this study and suggestions for health professionals, sports psychologists, and team counsellors are discussed.

Among various sports pursued in South Korea, volleyball has become one of the most popular sports leagues, resulting in high demand for international volleyball players. Hence, the migration of international sports players to South Korea’s professional leagues has been steadily increasing every year. According to the Korean Volleyball Federation (2018), the number of international volleyball players affiliated with South Korean professional volleyball leagues has reached 118 and is expected to continue to increase annually. With an increasing number of international volleyball players in South Korea, the sports industry and sports agencies must pay attention to the adjustment of the new players to a new society and culture.

According to acculturation theory, individuals who move to a new country experience both intercultural negotiations and social and psychological adaptations to new cultural values and beliefs (Berry, 1997). Prior studies have suggested that an adaptation process entails behavioural and attitudinal changes and challenges while interacting with culturally different individuals or groups (Berry & Hou, 2017; Berry et al., 1987Wang, Schwartz & Zamboanga, 2010). Such adaptation processes often cause migrants to experience cultural and racial conflicts, cultural misunderstandings, and intergroup anxiety (e.g., Dixon & Rosenbaum, 2004; Stonefish & Kwantes, 2017).

Multiple studies of acculturation have suggested that international sports players in South Korea experience various adaptation challenges that negatively influence their perceptions of their own health and wellbeing (Ahn & Kang, 2016; Chung et al., 2017). These adaptation challenges contribute to negative psychological symptoms (e.g., depression, anxiety), poor sports performances, and intentions to leave the Korean sports leagues (Ahn & Kang, 2016; Park & Choi, 2012). For example, Park and Choi (2012) reported that international sports players affiliated with the Korean leagues tend to experience high levels of isolation, frustration, and acculturative stress. To cope with these psychological problems, social scientists have sought effective strategies for helping international sports players adjust to their new society and culture in order to promote their health and wellbeing (Chung et al., 2017; Maeng & Cho, 2012; Park & Choi, 2012).

Previous findings have stressed the importance of leisure engagement as a way of facilitating acculturation and promoting social and psychological health among immigrants and migrants (e.g., Kim & Kim, 2013; Kim, Park et al., 2016). These studies have indicated that leisure engagement helps them develop friendships with host individuals, gain self-esteem and confidence, and acquire cultural knowledge and understanding. These positive benefits through leisure engagement can contribute to social and psychological health. For example, Jo et al. (2017) found that Chinese immigrants who engaged in soccer with host individuals developed cultural sensitivity and received social and emotional support, which improved their psychological wellbeing.

In addition, leisure engagement has been identified as one of the most effective coping strategies for immigrants and migrants to deal with acculturative stress (J. Lee et al., 2014; Li et al., 2015). Prior studies have suggested that, through leisure engagement, they build emotional and social support and access coping resources to deal with adaptation challenges. For example, Kim, Kim et al. (2016) found that participation in sport club activities allowed Korean immigrants living in the USA to embrace different cultural values, cope with acculturative stress, create ethnic strength, and experience personal growth.

Based on this evidence of the benefits of leisure engagement among immigrants and migrants, it may be presumed that international volleyball players also gain health benefits through leisure; however, little information exists on how their leisure engagement contributes to health benefits associated with acculturation. Although, due to their increasing numbers, multiple studies have examined the phenomenon of international sports players (Coakley & Dunning, 2000; Elliott & Maguire, 2008; Maguire, 1996), these have focused mainly on understanding motives for migration, migration experiences, and recruitment processes of international sports players who moved to Western cultures (Chung et al., 2017; Maeng & Cho, 2012; Park & Choi, 2012). Thus, it is important to further explore the value of leisure engagement among international volleyball players in more diverse national contexts. Thus, the purpose of this study is to capture the health benefits resulting from leisure engagement among international volleyball players affiliated with Korean volleyball leagues. This study can provide practical implications and suggestions to health professionals, sports psychologists, and team counsellors.

## Acculturation theory and leisure

Acculturation refers to a multifaceted and interactive process of cultural and psychological changes through cross-cultural contacts and interactions with culturally and ethnically diverse individuals (Gibson, 2001). According to Berry (1997), during the acculturation process, over time, immigrants are exposed to changes in beliefs, emotions, attitudes, values, behaviours, and social norms. The prevailing theory of acculturation is Berry’s (1980) bidimensional model, in which cultural maintenance and cultural acquisition constitute independent dimensions which determine four distinct acculturation outcomes (Corral & Landrine, 2008; Schwartz et al., 2010): (a) separation, (b) integration, (c) assimilation, and (d) marginalization.

More recently, Rudmin (2009) has suggested a new expanded acculturation model, based on a historical examination of acculturation constructs, which includes motivations for acculturation (e.g., cultural attitudes, ethnic identity, reaction to acculturative stress, and the cost and benefits of cultural learning); acculturative learning (e.g., information, instruction, imitation, mentors); and changes in individuals (e.g., skills, behaviours, cultural identification, social relations, beliefs, values). B. Kim and Abreu (2001) separated acculturation into behavioural, cognitive, and affective domains. Building on this approach, Schwartz et al. (2010) proposed that behavioural acculturation includes cultural practices, cognitive acculturation includes cultural values, and affective acculturation concerns cultural identification.

Acculturation theory has provided a solid foundation for investigations into immigrants’ processes of social and psychological adaptation to a new culture (Tsai et al., 2002). Within this line of inquiry, a number of studies have been focused on sports and leisure contexts, as acculturation has direct relevance to sports and leisure engagement among immigrants (e.g., Hatzigeorgiadis et al., 2013; Kim, 2012; Li et al., 2015; Li & Stodolska, 2006). The main findings of these studies show that leisure can facilitate the acculturation process and promote social and psychological health among immigrants. For example, Eitzen and Sage (2003) found that engaging in leisure activities helped immigrants socialize with mainstream society and facilitated their cultural integration into their host culture.

## Leisure behaviours and immigrants

Leisure scholars have confirmed the value of leisure engagement as a means of promoting health and quality of life among immigrants to South Korea (Kim et al., 2018; H. Kim et al., 2017). Previous studies have focused mainly on such leisure benefits for these immigrants as social support (I. Lee et al., 2012), physical and mental health (Kim et al., 2018), life satisfaction (Yi et al., 2011), and acculturation (Y. M. Kim et al., 2014). For example, Yi et al. (2011), based on interviews with immigrant workers living in South Korea, found that leisure activities served as an important vehicle for enhancing their life satisfaction and quality of life.

A growing body of literature has investigated the relationships among leisure, health, and acculturation (Ito et al., 2011; S. K. Lee et al., 2000; Kim & Iwasaki, 2016; Kim, Park et al., 2016; Zhou et al., 2018). These studies have shown that leisure engagement contributes to reduction of acculturative stress and increase in health among immigrants. By participating in leisure, immigrants have also gained cultural knowledge, developed cross-group friendships, and constructed effective coping strategies (Li et al., 2015; Seo et al., 2017; Walker et al., 2015). Participation in leisure activities has also been found to provide opportunities for immigrants to experience positive intergroup interactions, gain cultural understanding, and reduce negative feelings and acculturative stress (J. Lee et al., 2014; Li et al., 2015).

Other studies have investigated the relationship between types of leisure activities and health benefits among immigrants (Ito et al., 2011; Kim et al., 2018, 2014). For example, Ito et al. (2011) found that involvement in judo had a positive influence on Brazilians’ adaptation to Japanese culture by providing a vehicle of common understanding and language between judo partners that served to facilitate acceptance of each other’s cultural values and beliefs. In another study, Kim et al. (2018) found that participation in home-centred and social activities was positively associated with life satisfaction and health benefits among Western immigrants living in South Korea.

I. Lee et al. (2012) explored the benefits of participating in multicultural festivals in South Korea for international tourists and immigrants. They found that multicultural festivals provided ample opportunities for these individuals to gain cognitive, transformational, emotional, and social benefits. Similarly, Lee and Funk (2011) found that recreational sports played an important role in facilitating the assimilation and integration of Western immigrants to Korea. Furthermore, C. Lee et al. (2018) found that leisure engagement was an efficient way for non-Korean Asian international students to receive social and emotional peer support and gain a sense of belonging, which resulted in improved school adjustment.

## Methodology

### Participants

A purposeful criterion sampling strategy suggested by Patton (1990) was employed in this qualitative study. The criteria for the participants were that they (a) had moved to South Korea from a Western country, (b) had lived in South Korea at least one year, and (c) played on a Korean volleyball team. To recruit the study participants, the research team contacted the V-League teams and had meetings with directors and coaches affiliated with Korean volleyball teams. The research team obtained permission to distribute a brief introduction to the study, and afterwards interested parties contacted us via email or phone. Twelve American volleyball athletes voluntarily participated in the study. Twelve individuals ranging in age from 23 to 31 (M = 29.9) participated in the study, of whom seven were female. All were from the USA (See, Table 1). The average length of time since their migration was 16.5 months. The sample was determined sufficient on the basis of theoretical saturation as suggested by Mack et al. (2005). Approval from the university Institutional Review Board (IRB) was attained.

**Table 1.** Demographic characteristics of participants.

Name	Age	Gender	Length of Stay (month)	Educational Background
Ryan	31	Male	31	Master
Nikki	26	Female	14	Bachelor
Maria	27	Female	19	Bachelor
Tiffany	23	Female	9	Bachelor
Sarah	25	Female	14	Bachelor
Evan	28	Male	21	Bachelor
Marco	24	Male	13	Bachelor
Joel	25	Male	13	Bachelor
Milan	23	Female	9	Bachelor
Matthew	26	Male	21	Bachelor
Eva	28	Female	21	Master
Jeff	24	Male	13	Bachelor

### Interview protocol

A semi-structured interview procedure was utilized to elicit the participants’ perceptions of the leisure benefits of participating in Korean volleyball leagues. Each player’s interview was conducted in English and ranged from approximately 30 to 60 minutes in duration. The interviews took place between March and August 2017. Content-mapping and content-mining questions as suggested by Legar, Keegan, and Ward (2003) were employed. The content-mapping questions were used to explore the broad immigration experiences of each participant. Examples included “When did you move to South Korea?” and “Could you tell me about your life story?” The content-mining questions were asked to capture detailed descriptions of experiences related to leisure, such as “What do you do when you have free time?” “Why do you participate in the activities you mentioned?” “What benefits do you experience when participating in these activities?” and “What role, if any, has these activities had in helping you deal with the challenges in your life after you moved to South Korea?” All of the interviews were recorded and transcribed verbatim, and the data were analysed using analytic induction and comparative procedures to identify common themes in the participants’ responses (Strauss & Corbin, 1998).

### Data analysis and trustworthiness

To capture the participants’ perceptions of the benefits of leisure activities, Creswell’s (2009) five steps of qualitative data analysis were followed: (a) creation of raw data (e.g., transcriptions, field notes), (b) data organization for analysis, (c) exploration of data, (d) generation of general themes with direct quotes, and (e) data interpretation.

1. After each interview, the research team generated a transcript and made preparations for data analysis. The first author distributed the complete transcripts to each study investigator along with the raw data sets.
2. Each study investigator read every transcript independently and thoroughly.
3. The research team explored every transcript and created/identified general themes for each one.
4. The research team reorganized the general themes/subthemes, noting relevant direct quotes on each transcript.
5. The research team compared the labelled themes and interpreted them iteratively comparing all transcripts. When consensus was reached on interpretations of all data sets, the final set of themes and subthemes were produced.

The data were analysed and coded using the constant comparative method (Merriam, 1998), which allowed the research team to sharpen their focus and refine their analysis until reaching the saturation point.

The research team utilized two methods to increase the credibility of the analysis. First, a member-checking was used to allow participants to verify the researchers’ interpretations of the data from the participants. The research team followed the procedures proposed by Peterson et al. (2007), by which the participants were invited to review a summary of the themes identified. This method is based on voluntary participation, and the eight participants who engaged in this member-checking process expressed satisfaction with the data interpretations. In addition, all members of the research team specialized in qualitative studies, which supported the trustworthiness of the analysis, and each member interpreted the data and generated themes independently, following which the team negotiated consensus on the final data interpretation.

## Findings

Based on the participants’ statements and experiences, it was determined that their new leisure environment affected their leisure choices and preferences. All of the participants mentioned that they encountered a variety of adaptation challenges, such as language barriers, cultural and ethnic differences, and a lack of social support. Such challenges generated acculturative stress and feelings of loneliness and isolation. Due to communication differences with their South Korean teammates, the participants also expressed some challenges related to team-based communications, including expressing thoughts and opinions in a culturally appropriate manner.

On the positive side, the participants identified their favourite leisure activities as physical activities (e.g., running, Taekwondo), listening to music, yoga, social activities/social gatherings, language exchange programme, travelling to historical and cultural sites, shopping, running, and other recreational activities. Three major themes were identified pertaining to the benefits of these leisure activities: (a) new leisure opportunities and coping, (b) inter-intra group friendships, and (c) acculturation. These benefits can contribute to social and psychological health, resulting in an increased quality of life.

### New leisure opportunities and coping

According to most of the participants, their leisure activities played an important role in avoiding or reducing stress and helped them cope with negative psychological states. For example, Ryan (male, 31) mentioned that his Taekwondo practice improved his ability to find inner peace and patience, which helped relieve stress. He also volunteered to teach Taekwondo to other foreigners and mentioned that his volunteer experience helped him deal with his adaptation challenges. Others expressed similar experiences with the beneficial effects of leisure activities with such statements as the following: “Whenever I am stressed out, I run.” “Drawing helps reduce my stress.” “Traveling was a good way to deal with my stress.” Nikki (female, 26) found practicing new dance techniques beneficial: “I have been learning hip pop dance since I have been here. It has helped me a lot as I feel like, physically, I am good and, also, it definitely reduced my stress.”

Some of the participants pursued new forms of leisure activities that helped reduce their stress, such as hot yoga, running or walking through parks, and visiting stores and shops in the evening, which was a novelty because safety issues prevented them from walking or shopping at night in their own countries. The new leisure activities they embraced after moving to South Korea helped them reduce their stress and refocus on their volleyball games. For example, Maria (female, 27) said,

I never expected to go running at night in my home town back in the states. Running at night really helped me focus on my games and totally got rid of my stress. Believe it or not, I was running at night five times a week.

Such new experiences could be invigorating as well as calming, resulting in redirection of energy from stress to performance.

Some of the participants mentioned that they enjoyed relaxation at Jimjilbang, a large gender-segregated public bathhouse equipped with a sauna and recreational games. They described Jimjilbang as an effective place for stress reduction. In addition, a few of the participants who engaged in Taekwondo and hot yoga stated that besides unique Korean movements and skills they learned how to focus on mindfulness and composure. As a result, they gained confidence, which improved their self-esteem and helped reduce their stress levels.

These examples indicate that the participants used leisure as a way of dealing with stress. Exploring and engaging in new forms of leisure activities improved their self-confidence and self-esteem. Such engagement in leisure served as an important vehicle for reducing stress.

### Inter-intra group friendship

All of the participants reported that leisure activities helped them maintain intragroup friendships and establish intergroup friendships, which gave them social and emotional support. In terms of intragroup friendships, they experienced enjoyment in their interactions with other immigrants from Western countries, with whom they shared immigration experiences as well as similar cultural backgrounds. For example, Tiffany (female, 23) spent leisure time travelling with a group of other immigrants from England and Australia, with whom she bonded and from whom she received emotional and social support.

In addition, after their volleyball games and practices, participants formed unique friendships with other international volleyball players on the other teams. When they competed against other South Korean teams, making friends seemed difficult because of the competition. But off the court they found they could lower the barriers and relate to opposing team members as friends rather than rivals. According to Ryan (male, 31),

When we played, we were so competitive and wanted to win each game … it was not usual to hang out with other team players. However, we played here as professional volleyball players and became good friends because we thought we represented the U.S. as a group.

In this and other ways, the participants found that leisure activities produced a context in which they could form intergroup friendships with Koreans. Initially, they found it was difficult to make Korean friends because of the language barrier and cultural differences. However, by participating in leisure activities, they were given rich opportunities to socialize with Koreans and create friendships. They discovered cultural similarities as well as differences through leisure engagements that resulted in intergroup friendships.

Some of the participants became involved with culture-related activities. For example, Sarah (female, 25) and Evan (male, 28) mentioned that their favourite activity was to go to Jjimjilbang, which was open 24 hours and a great weekend getaway for them. Similarly, Marco (male, 24) said,

I really hung out with my Korean friends to Noraebang (a singing venue, where they danced and sang together). I enjoyed dancing and singing with them … it was so much fun!

A few of the participants were involved in language partner programs that allowed them to teach each other their native languages. For example, Milan (female, 23) stated that she actively engaged in a language partner programme in which she taught English to her partner and her partner taught her Korean language and culture. Through this program, they expanded their social networks and developed intergroup friendships.

These examples show that through leisure activities the participants created friendships both with others who had similar cultural backgrounds and with Koreans and pursued enriching culture-related activities. Based on these findings, it can be seen that leisure engagement provides rich opportunities for participants to expand their social networks and create social bonding.

### Acculturation

The participants also reported that leisure activities facilitated their acculturation processes by allowing them to gain cultural knowledge and develop cultural understandings. They shared that they were given opportunities to explore different aspects of their new culture through leisure engagement. For example, most of the participants learned the significance of age, and in communicating with others with the Korean culture, in particular, the deference expected to be paid to elders with whom they communicated, as well as the respect paid to elders’ views when a group decision was made. According to Joel (male, 25), he learned that the oldest person in his Taekwondo activity significantly influenced decision-making. Based on this experience, he acknowledged the importance of age in his interactions with his Korean volleyball teammates. In addition, Marco (male, 24) stated,

One thing I learned about Korean culture is to use two hands to give something to others who are older than me. My friend explained to me that in their culture using two hands is a sign of respect.

In a similar way, the participants acquired valuable information and knowledge related to the Korean culture, customs, and traditions by visiting historical and cultural sites. These activities allowed them to improve their cultural sensitivity and gain cultural and historical understanding. Participants also emulated aspects of Korea’s urban lifestyle. Milan (female, 23) said that she embraced the “hurry up” culture her Korean friends demonstrated when they travelled or participated in other activities and created her own fast-paced lifestyle which she practiced in her daily routine.

The participants perceived the collectivistic values and beliefs ingrained in Korean culture. When they participated in activities, they observed that Koreans went out as a group and stayed together the whole time. When they had free time before and after games, they were most likely to be together socializing. When they visited restaurants, the Korean players shared their dishes with each other as part of their culture. Mathew (male, 26) said that he appreciated the Korean culture of sharing as it instilled a sense of belonging and strengthened the team.

The experiences the participants described indicated that leisure engagement helped them to gain cultural knowledge and embrace Korean culture. Based on their cultural growth and willingness to learn, they applied their experiences to their sportsmanship and communications with other team members, which they felt benefitted them both personally and as players.

## Discussion

This qualitative study explored the health benefits of leisure engagement for international volleyball players in Korea. The findings of this study showed that participation in leisure activities facilitated the participants’ acculturation, improved their mental health, and provided social benefits. This study suggests that leisure provides rich opportunities for participants to explore new experiences, deal with stress, gain cultural knowledge and understandings, and develop friendships with others. This study thus indicates that the benefits of leisure engagement contributed to these international sports players’ social and psychological wellbeing. In addition, these benefits helped the participants communicate better with their peers and focus on their professional sports performances.

Prior studies have shown that immigrants living in Western countries have used leisure as a way of developing coping strategies (e.g., J. Lee et al., 2014; Li et al., 2015). These studies have suggested that leisure served as a coping mechanism that helped immigrants deal with acculturative stress. The findings of the current study are aligned with these studies in that the international volleyball players also used leisure as a resource for developing coping strategies. In particular, this study suggests that exploring and engaging in new forms of leisure activities can be an important resource in regard to coping with stress and promoting mental health. In addition, this study suggests that the coping abilities developed through leisure experiences and intra-inter group friendships can result in improved confidence and self-esteem. The results of this study indicate that leisure can serve as an important function in developing coping strategies and promoting mental health in international volleyball players.

Multiple empirical studies have supported the idea that participation in leisure activities facilitates acculturation among immigrants (Stodolska & Alexandris, 2004) by helping them gain cultural knowledge, socialize with host individuals, and develop cultural understandings (Li & Stodolska, 2006; Stodolska & Yi, 2003; Yu & Berryman, 1996). The current study expands this body of knowledge by focusing specifically on international volleyball players who play in South Korea. In particular, culturally meaningful activities, such as Jimjilbang, Taekwondo, and cultural tourism, may serve as important vehicles for facilitating acculturation among international volleyball players by allowing them to embrace new cultural perspectives, gain cultural knowledge and understandings, and develop intergroup friendships.

One striking implication from the perspectives of intergroup contact theory and social identity theory in the present study is that the international volleyball players needed to actively engage in leisure activities both with in-group and out-group members for successful acculturation. When international volleyball players integrate the necessary information of several significant stressors and solve problems in leisure contexts, they can establish or re-establish control, which ideally culminates in adaptation in a culturally different country. Some researchers have incorporated intergroup contact theory, suggested by Pettigrew and Tropp (2006), into leisure and recreation settings (Kim, 2012; Kim et al., 2015). They suggested that Asian immigrants used leisure engagement as a way of establishing intergroup friendships, gaining cultural knowledge and understandings, and experiencing positive attitudes towards other racial groups. The findings of the current study suggest that leisure engagement helped the international volleyball players to form inter -group friendships. Thus, this study confirms the intergroup contact theory, which emphasizes the value of positive intergroup contact and interaction among multi-ethnic groups as well as host country people.

In addition, previous studies have found that immigrants have a tendency to participate in leisure activities with others who have similar cultural backgrounds (Elling et al., 2001; Walseth, 2006), presumably because those people have more positive attitudes towards and stereotypes of in-group members than out-groups. Social identity theory states that individuals tend to interact with others who have similar cultural and ethnic characteristics and tend to express in- and out-group biases (Tajifel & Turner, 1986). Consistent with previous studies (e.g., Kim, Park, et al., 2016), the international volleyball players had a tendency to develop favouritism and a sense of friendship with other in-group members and to maintain cultural identification and membership. That is, enjoying leisure with in-group members facilitates feelings of belonging, comfort, a positive social identity, and a sense of friendship. Walseth (2006) found that in sports, in-group members are likely to create cultural bonding and a social and emotional support system that contributes to in-group cohesion. The present study also supports this idea as the international volleyball players tended to form intra-group friendships and bond with other players who had similar cultural backgrounds and shared similar migration experiences, which resulted in in-group bonding.

From a theoretical perspective, the current study supports Rudmin’s (2009) acculturation model by suggesting that the adaptation process through leisure engagement is multidimensional as it includes motivations for acculturation, acculturative learning, and changes in individuals. The findings of this study suggest that international volleyball players can experience multifaceted and continuous processes of embracing and negotiating new aspects of culture through leisure engagement. From a practical point of view, staff members and/or mental health counsellors of professional volleyball teams who oversee international players need to identify each player’s needs and wants, as well as their difficulties from the perspective of acculturation. Further, they need to design individualized leisure program based on this assessment. For instance, if an international player faces difficulties in cultural adaptation, engagement in a Taekwondo program can be effective for learning Korean culture and making friends.

## Limitations and future studies

This qualitative study explored only a small group of international volleyball players who had moved to South Korea. Other international players who have moved to Western countries also experience new leisure pursuits and leisure challenges associated with acculturation. To further investigate cross-national acculturation, future studies might explore study of leisure, acculturation, and health among international players from Eastern and Western cultures in other settings. In addition, international players in Korea who are from other Asian countries may have different levels of acculturation and intergroup contact through leisure activities, perhaps because they are generally familiar with Eastern cultural values and beliefs, social norms, and leisure activities. In the future, exploring how international sports players’ cultural backgrounds can influence leisure behaviours and associated health benefits can provide insightful information to sports professionals.

Future research analysing the acculturative process of international volleyball players through leisure will benefit from the use of longitudinal designs with larger sample sizes. In addition, research efforts should be made to examine the leisure benefits of different professional sports and their unique cultures. The current study may contribute to the development and implementation of practical leisure programs for international sport players in consideration of their acculturation, health, performance, and well-being. In addition, other important variables may influence the leisure behaviours of international sports players, including the length of their stay in a given country, their levels of acculturation, and their past leisure experiences. These factors can affect leisure choices and associated health benefits. Future studies are needed to explore the relationships among leisure, acculturation, and health among international sports players from various countries and in various host settings. They can provide practical suggestions on how leisure can facilitate players’ acculturation and provide health benefits.

## Implications and conclusion

Professional teams and their managers need to provide international sports players with a variety of leisure resources and opportunities. By providing resources and information related to leisure in host countries, teams can maximize the leisure options of international sports players and the benefits drawn from leisure participation. In addition, leisure professionals need to design and implement new leisure and recreational programs so that international sports players have access to a wide range of leisure and recreation opportunities. This will encourage international sports players to explore more leisure options and gain better cultural knowledge and understanding.

Health professionals need to create recreational programs, such as multicultural games and events, that foster positive intergroup contact and interactions between international sports players and members of their host nation. As demonstrated in this study, by participating in recreational programs with Korean individuals, international sports players were given opportunities to establish and develop friendships with these individuals, understand new aspects of Korean culture, and examine their own culture.

This study stressed the importance of leisure engagement as a way of promoting social and psychological well-being among international sports players. The findings of this study suggest that leisure can facilitate an individual’s acculturation, improve their mental health, and provide social benefits. Designing and implementing appropriate leisure programs and activities are necessary to help international sports players focus on their games and gain health benefits.
